# New variants of ALMS1 gene and familial Alström syndrome case series

**DOI:** 10.1016/j.bjorl.2024.101402

**Published:** 2024-02-22

**Authors:** Isabela Carvalho de Queiroz, Natália Carasek, Luiza Costa Villela Ferreira, Lucas Alves Teixeira Oliveira, Fernando Massa Correia, Thaís Gomes Abrahão Elias, Fayez Bahmad

**Affiliations:** Universidade de Brasília (UnB), Brasília, DF, Brazil

**Keywords:** ALMS1, Alström syndrome, Sensorineural hearing loss, Ciliopathy

## Abstract

**Objectives:**

To report two new variants of ALMS1 gene and to discuss the audiological evolution and clinical phenotype in two pairs of siblings with Alström syndrome.

**Report:**

This paper is a multi-disciplinary diagnostic evaluation, with genetic and audiological analysis that aims to report two new variants of the ALMS1 gene and to discuss the audiological evolution and clinical phenotype in a case series of patients with familial Alström syndrome. Therefore, we describe 4 cases presenting a complete audiometric profile of two pairs of unrelated siblings, to provide a better understanding of this very rare disease. Additionally, the present study identified two heterozygous mutations in the ALMS1 gene.

**Conclusion:**

This Clinical Capsule Report highlights the importance of audiological monitoring throughout the development of patients with Alström syndrome. The two variants found were not previously reported in the literature, which expands the spectrum of ALMS1 variants in Alström syndrome.

## Highlights

• New variants of the ALMS1 gene.
• Clinical phenotype associated with mutation.
• Audiological evolution of the reported patients.

## Introduction

In 1959, Alström et al.^1^ described a syndrome with retinal degeneration associated with obesity, diabetes mellitus and sensorineural hearing loss. Named as Alström syndrome (OMIM 203800), this rare, recessive, monogenic hereditary disorder is characterized by a complex set of clinical manifestations, usually starting in the first year of life. It affects several organs, including cardiac, hepatic, renal and pulmonary systems.^2^

The prevalence of this syndrome is estimated at 1–9 cases per million individuals,^3^, ^4^ with approximately 1053 cases reported worldwide.^5^ Its etiology was described for the first time in 2002, when Hearn et al.^6^ reported that the genetic aberration that caused Alström syndrome was a mutation in a new gene of unknown function, ALMS1, present on the chromosome 2p13. The structural modification was identified as a balanced reciprocal translocation [46, xy, +(2; 11)], (p13; q21). It is currently known that this gene encodes the ciliary protein ALMS1,^4^, ^7^ present in the centrosome, basal bodies of cilia, cytoplasm, cytoskeleton and microtubule organization center.^3^, ^8^

The ALMS1 protein is widely expressed in fetal and postnatal tissues and, despite not having its function completely elucidated, it seems to help the formation and maintenance of cilia, cell cycle regulation, endosomal traffic, cell migration and production of extracellular matrix.^9^, ^10^ Thus, Alström syndrome (AS) can be considered as a disorder belonging to the group of ciliopathies, such as Bardet–Biedl syndrome and Usher syndrome.^2^, ^9^

Individuals with Alström syndrome may have truncal obesity, hyperinsulinemia, type 2 diabetes mellitus, acanthosis nigricans, short stature, visual and hearing losses, dilated or restrictive cardiomyopathy with congestive heart failure, hypothyroidism, and hypogonadism.^11^ The onset of the characteristic signs and symptoms of the syndrome are progressive and non-simultaneous. Due to the broad spectrum of manifestations, early diagnosis is challenging.^4^ Therefore, we hereby describe and analyze the clinical presentation and audiological outcomes of 4 patients with the syndrome, describing their genetic patterns, including the description of new variants of the gene discovered in our research. The aim of this case series report is to deepen the knowledge on confirmed cases of the syndrome and the different possible presentations, helping the early recognition and treatment of these patients and improving their prognosis. Furthermore, the report of two previously unknown new variants of the ALMS1 gene is of the utmost relevance for further studies of the syndrome.

This study was conducted in accordance with the Declaration of Helsinki and written informed consent was obtained from the patients and/or their parents to write and submit this paper, alongside with the images and test results. Ethical review and approval were not applicable because this manuscript is a case series report that describes the steps in clinical care of a patient, written after the fact. No experiments were carried out with this patient.

## Case series report

### Patient 1

Male, currently 44-years old, followed by our team since the age of 30. This subject presented with photophobia since birth, nystagmus since 6-months old and retinitis pigmentosa from 10-months old. He also reported obesity in his childhood. Audiological examinations performed at the age of 6 showed moderate bilateral sensorineural hearing loss, initially attributed to unknown genetic etiology.

**Figure 1:**
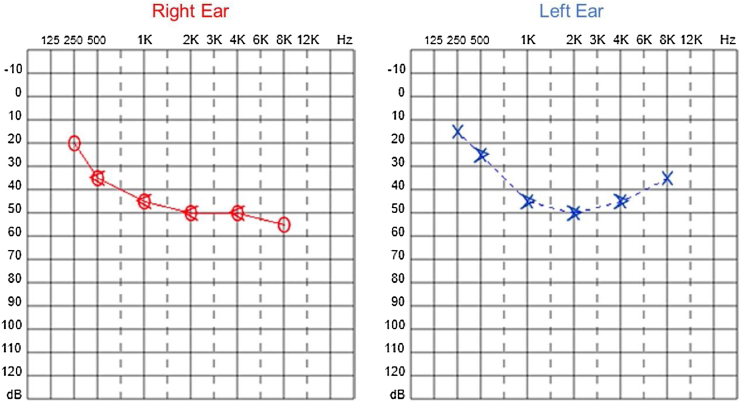
Audiometry of patient 1, 21-years old.

**Figure 2:**
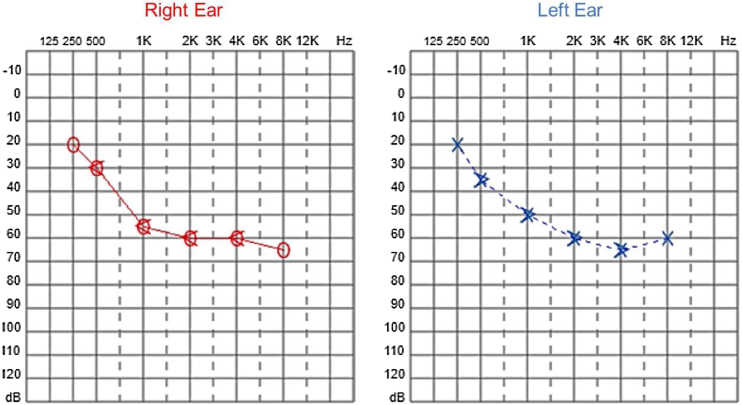
Audiometry of patient 1, 30-years old.

**Figure 3:**
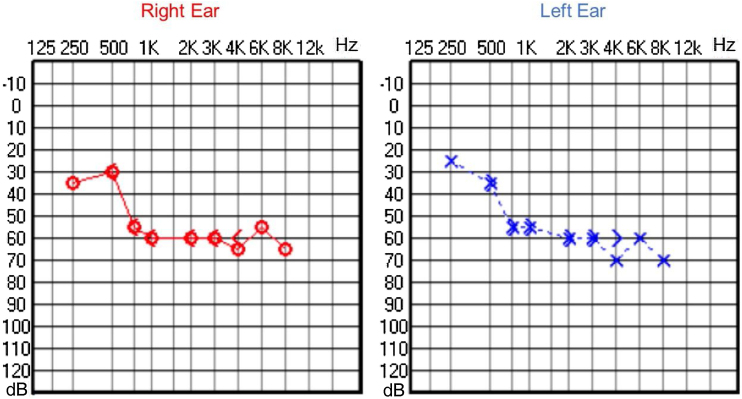
Audiometry of patient 1, 44-years old.

Since then, the patient was submitted to a series of tonal and vocal audiometries, which characterized his hearing loss as slowly progressive, with a descending audiometric curve. Figure 1, Figure 2 show the results of pure tone audiometry at ages 21 and 30, respectively. His most recent audiometric assessment, performed at 44-years old, is shown in Fig. 3. Tympanometry showed type “A” curves for both ears, in all assessments.

Otoacoustic Emissions (OAE) were absent in all frequencies from 2000 to 5000 Hz and the most recent Auditory Brainstem Response (ABR), performed at 90 dB HL, demonstrated the presence of the 5th wave bilaterally, with an interaural difference of less than 0.3 ms.

At the age of 27, he was diagnosed with diabetes mellitus, insulin resistance, hyperinsulinemia, hypertriglyceridemia, hypogonadism, hypothyroidism, and acanthosis nigricans. At 28-years old, he was diagnosed with dilated cardiomyopathy without congestive heart failure. The patient had no history of recurrent otitis media and so far have not had pulmonary, hepatic or renal symptoms.

### Patient 2

Male, currently 39-years old, brother of patient 1, assisted by our team since the age of 26. This subject presented with photophobia since birth and nystagmus developed by 6-months old. At 2-months, he was diagnosed with dilated cardiomyopathy, without congestive heart failure, treated for one year, with resolution of the condition. He had retinitis pigmentosa and obesity since childhood.

The patient had a history of recurrent otitis media since early childhood and developed liver dysfunction in his twenties but had no pulmonary or renal symptoms. Despite not having diabetes and acanthosis nigricans, the patient had hyperinsulinemia, hypertriglyceridemia, hypogonadism, and hypothyroidism.

**Figure 4:**
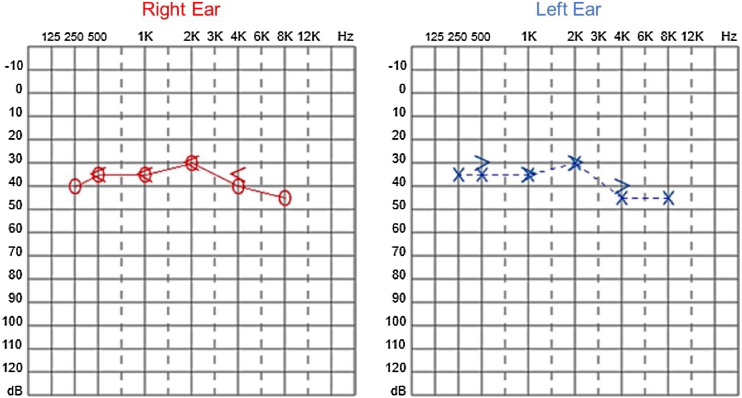
Audiometry of patient 2, at 11-years of age.

**Figure 5:**
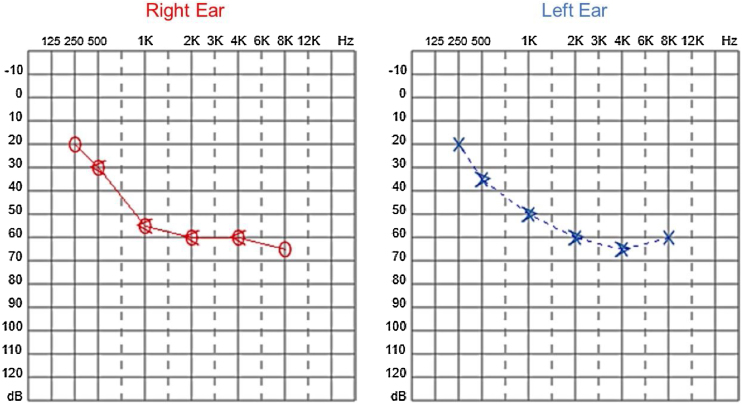
Audiometry of patient 2, 26-years-old.

**Figure 6:**
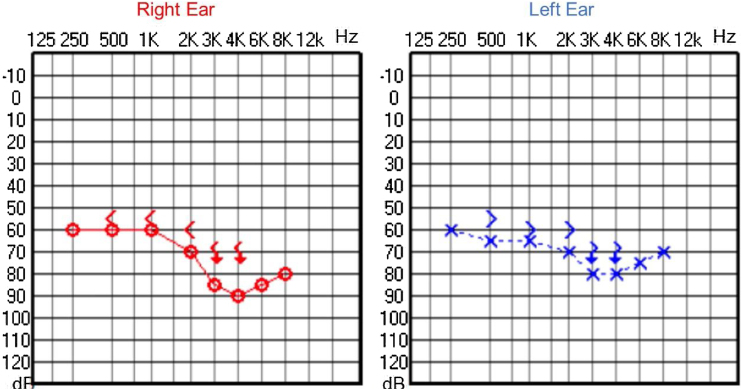
Audiometry of patient 2, 39-years-old.

Audiological examinations performed at 6-years old demonstrated bilateral sensorineural hearing loss, which was proven to be slowly progressive, as seen in the following audiograms. Figure 4, Figure 5 show the results of pure tone audiometry at ages 11 and 26, respectively. The most recent audiometric evaluation, at 39 (Fig. 6), detected a moderately severe sensorineural hearing loss, with bilateral descending configuration. Tympanometry had type “As” curves in both ears. Distortion Product Otoacoustic Emissions (DPOAE) in the frequencies from 1500 to 6000 Hz were bilaterally absent in the latest evaluation and the most recent ABR demonstrated the absence of waves I, III and V.

**Figure 7:**
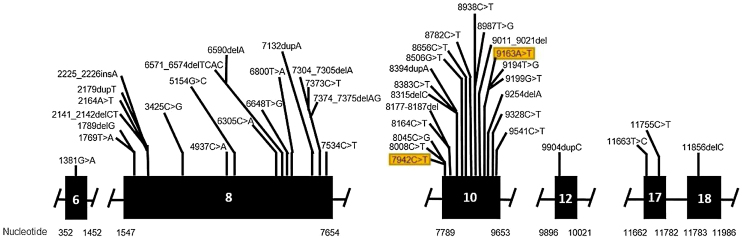
Mutation in the ALMS1 gene.

**Figure 8:**
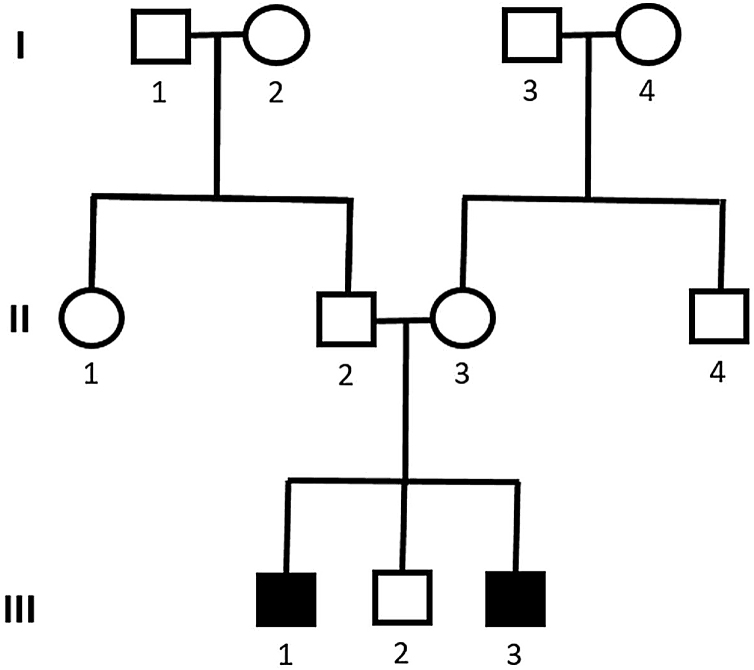
Pedigree showing cases 1 and 2 with Alström syndrome (case 1: III-1 and case 2: III-3).

Genetic analysis of patients 1 and 2 confirmed the mutation in the ALMS1 gene in both brothers. Two heterozygous mutations were identified in exon 10 of ALMS1 (c.7942C>T, Gln2648* and c.9163A>T, Lys3055*), confirming the diagnosis of Alström syndrome (Fig. 7). The pedigree chart illustrates these findings in Fig. 8.

### Patient 3

Female, born by cesarean delivery, at 40-weeks of an uneventful pregnancy. At 33-days old, after a crisis of cyanosis while breastfeeding, the patient was diagnosed with dilated cardiomyopathy. At the age of 2, she began to present nystagmus, when the diagnosis of Alström syndrome was made. By 3-years old, obesity symptoms settled in, by the time she was first evaluated by our team.

**Figure 9:**
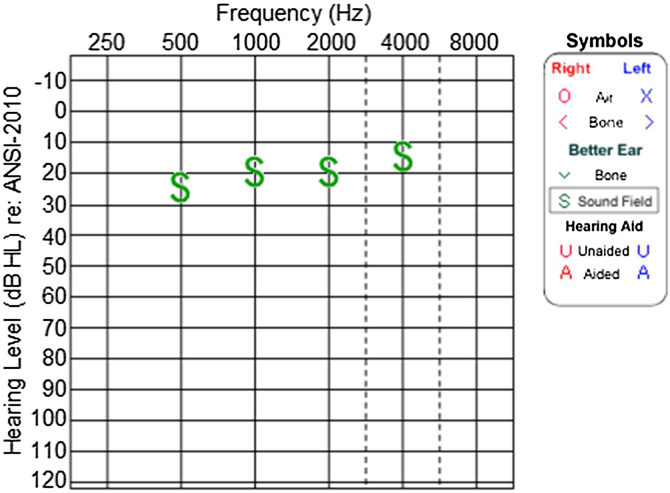
Audiometry of patient 3, at 3-years and 4-months-old.

**Figure 10:**
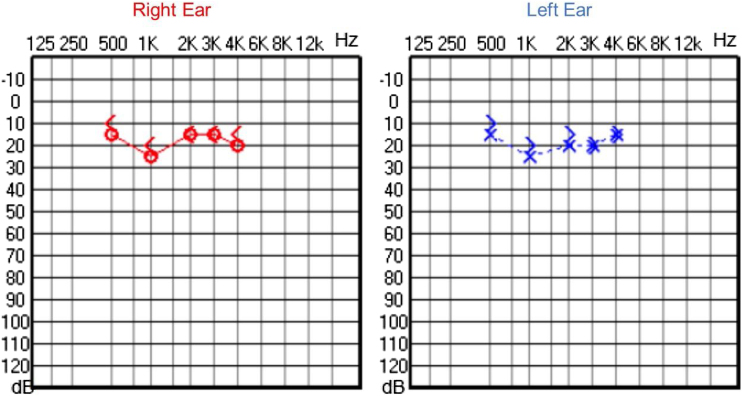
Audiometry of patient 3, at 4-years and 10-months of age.

Audiological evaluation at 3-years and 4-months, through Audiometry performed in free field with visual reinforcement (Fig. 9), and at 4-years and 10-months through Visual Reinforcement Audiometry (VRA) with headphones in frequencies from 500 to 4000 Hz (Fig. 10), indicated mild bilateral sensorineural hearing loss. Tympanometry was normal, with type “A” curves in both ears. OAE were present and ABR waves were normal in all evaluations.

At the age of 4 she was also diagnosed with low vision, beginning to use corrective lenses. Currently at 5 years old, the Body Mass Index (BMI) is above the 97th percentile, according to curves developed by the World Health Organization (WHO) in 2007, which consider the cutoff points for overweight and obesity to be the 85th and 97th percentiles, respectively.^12^, ^13^ As for now, she has not had hyperinsulinemia, diabetes or hypothyroidism, as well as gastrointestinal, genitourinary or respiratory symptoms. There is no evidence of cognitive or language deficits, nor autism spectrum behaviors.

### Patient 4

Male, brother of patient 3, born by cesarean delivery, at 40-weeks of an uneventful pregnancy. He developed mild dyspnea at 2-months, being diagnosed with dilated cardiomyopathy, with a subsequent diagnosis of Alström syndrome. He was first evaluated by our team at 1-year and 3-months. Currently, at the age of two, he has a BMI compatible with childhood obesity (above the 97th percentile for his age).^14^, ^15^ The parents did not perceive signals of hypoacusis, but noticed some autistic spectrum behaviors, also with delay in language and global development. To date, he does not present nystagmus, hyperinsulinemia, diabetes, hypothyroidism, gastrointestinal, genitourinary, or respiratory symptoms.

**Figure 11:**
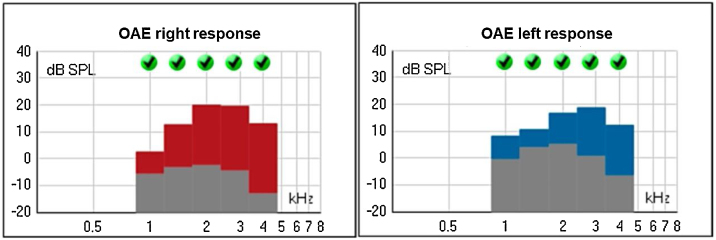
Transient Evoked Otoacustic Emissions (OAE) of patient 4, at 2-years of age.

The Transient Evoked Otoacustic Emissions (TEOAE), performed at two years old, were present with good amplitude, suggesting the integrity of the outer hair cells and enabling the inference of thresholds better than 25 dB (Fig. 11).

**Figure 12:**
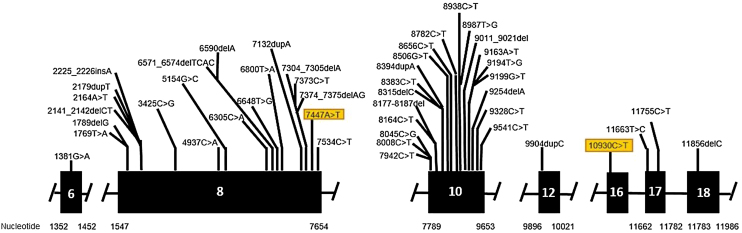
Mutation in the ALMS1 gene.

**Figure 13:**
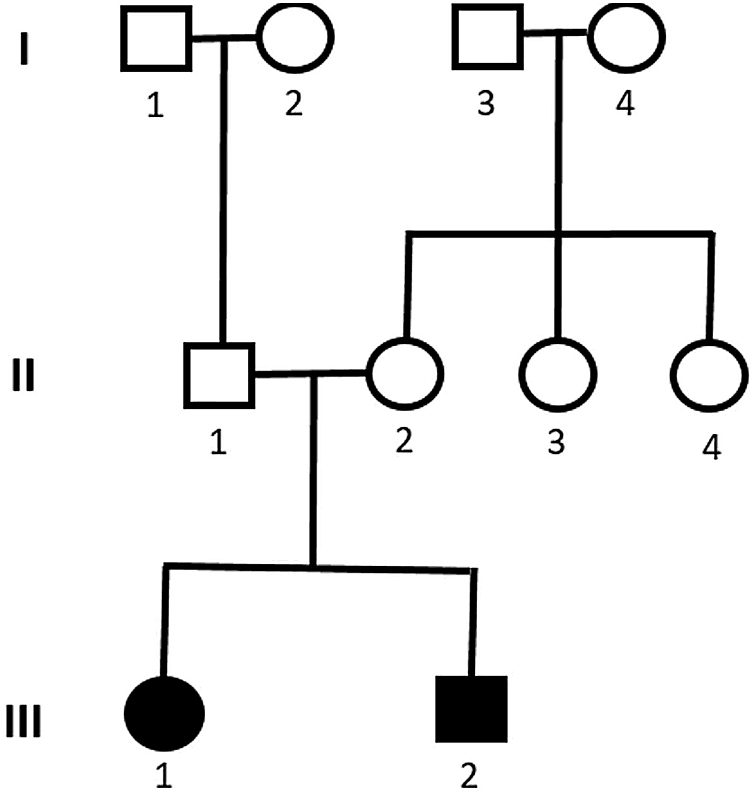
Pedigree showing cases 3 and 4 with Alström syndrome (case 3: III-1 and case 4: III-2).

Genetic analysis of the patients 3 and 4 confirmed the mutation in the ALMS1 gene in both brothers. Two heterozygous variants were found in the ALMS1 gene: c.7447A>T, p.Lys2483* and c.10930C>T, p.Gln3644*, confirming the diagnosis of Alström syndrome (Fig. 12). These variants, to our knowledge, have not been previously described in the medical literature.^14^ The pedigree chart (Fig. 13) synthesizes these findings.

## Discussion

Hearing loss is the most common type of sensory impairment and there are several possible etiologies. Non-hereditary causes include infectious, autoimmune, traumatic, and vascular causes. Hereditary causes can be syndromic or non-syndromic, depending on the association or not, respectively, of other phenotypic alterations.^15^ The so-called syndromic forms are responsible for approximately 30% of cases of deafness in children.^15^ Alström syndrome is part of this group, with the patient presenting, among other phenotypic characteristics, an early onset progressive sensorineural hearing loss.

In our case series, four members of two unrelated Caucasian families were evaluated. Two members of the same family had slowly progressive bilateral sensorineural hearing loss from the age of 6. Currently, at 44 and 39-years old, these patients have moderate and moderately severe sensorineural hearing loss, respectively, both rehabilitated with hearing aids bilaterally.

The hearing in the other two cases, brothers from another family, was also assessed. Patient 3 began to present hearing complaints at 3-years and 4-months old, with mild bilateral sensorineural hearing loss being evidenced in the audiogram. Patient 4, currently 2-years old, does not have any hearing deficits so far. These findings are consistent with the results by Marshall et al.,^4^ who demonstrated that sensorineural hearing loss can start between the ages of 2 and 21-years in the syndrome. In patients 1 and 2, EOA were absent (signal/noise ratio below normal), while tympanometry and ABR were normal. These results suggest cochlear damage, compatible with histopathological findings of Corti’s organ degeneration, published by Nadol et al.^16^

The main differential diagnosis of AS is Usher’s syndrome, which also presents with hearing loss of early onset and slow progression, associated with visual loss.^17^ The association with other phenotypic alterations in AS (such as the presence of type 2 diabetes mellitus, hypertriglyceridemia, hypogonadism, hypothyroidism, renal and hepatic dysfunction and childhood obesity) can help in the differential diagnosis of these two entities, even though they manifest themselves in a variable way among the patients and years.

**Figure 14:**
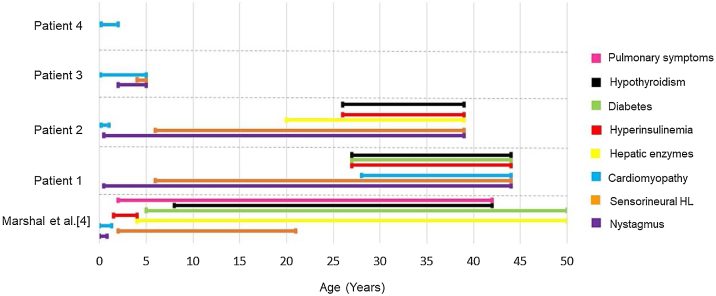
Age of onset for each clinical presentation of Alström syndrome in the cohort of Marshall et al.^4^ compared to the appearance of symptoms in this study.

In our study, other phenotypic aspects were evaluated and compared with the results previously described by Marshall et al.^4^ (Fig. 14). The authors^4^ had found that 98% of patients had nystagmus, starting between birth and 40-weeks of life. Our patients 1 and 2 also presented nystagmus in this timeframe, at 6-months. Patient 3 developed nystagmus at two years old, while patient 4 has not developed this symptom so far. When evaluating the presence of hyperinsulinemia, Marshall et al.^4^ observed that it usually develops between the ages of 18-months and 4-years old (92%), while diabetes mellitus usually occurs between 5 and 50 years old, with an average age of onset at 16. In our study, 50% of the patients had hyperinsulinemia, with onset only in adulthood, and only one patient (25%) has been diagnosed with diabetes mellitus so far, with fist symptoms at 27-years old.

Cardiomyopathy, one of the cardinal symptoms of AS, is probably underestimated in its prevalence, as some babies succumb to heart failure before the diagnosis of Alström syndrome. In the study by Marshall et al.,^4^ 43% of the patients had cardiomyopathy in the childhood, with onset between 1-week and 16-months of life, while 18% developed late dilated cardiomyopathy, between 7 and 32-years old. In our study, we found three subjects (patients 2–4) who presented with childhood cardiomyopathy. Only one case (patient 1) developed cardiomyopathy in adulthood, at 28-years old. These findings are consistent with those found in other literature reports as well.^4^, ^5^, ^18^, ^19^, ^20^

Hypothyroidism occurs in 20%–30% of patients with AS,^4^, ^21^ with onset varying between 8 and 42-years.^4^ The occurrence of hypothyroidism in our patients was also consistent with the literature, with two cases (patients 1 and 2) presenting this alteration at 27 and 26 years, respectively. Elevated liver transaminases can occur in variable levels in AS^22^ and the severity of the liver damage in these patients is significantly greater if compared to equally obese controls.^21^ Unlike Marshall et al.,^4^ who found elevation of transaminases as early as 4-years of age in 89 of 97 patients (92%), our study found liver alteration in only one case (patient 2), starting at 20-years-old.

Pulmonary involvement severity varies from frequent respiratory infections to pulmonary fibrosis and pulmonary hypertension.^22^ Lung symptoms are reported in approximately 50% of patients,^4^, ^23^ in an age range of onset between 2 and 42-years.^4^ In this study, however, no patients presented pulmonary symptoms so far. Fig. 14 is a graph illustrating the age of appearance of each Alström syndrome symptom in the cohort of Marshall et al.^4^ compared to the patients in this study. All of our patients undergo multidisciplinary follow-up, according to the clinical manifestations they present, with periodic otorhinolaryngological, endocrinological, cardiological and ophthalmological evaluations, among others as needed. Specific medications are administered in each case as necessary, depending on the clinical manifestations.

Molecular genetic analysis must be used for diagnostic confirmation in AS. Although currently there is no preventive approach, it is important to establish the genetics of the sensorineural hearing loss as soon as possible, aiming for adequate family counseling, appropriate management of medications and social or educational adjustments that can compensate for the multiple deficiencies.^24^ Genetic analysis of our patients confirmed the ALMS1 gene mutation in all of the cases. In patients 1 and 2, two heterozygous mutations were identified in exon 10 of ALMS1 (c.7942C>T, Gln2648* and c.9163A>T, Lys3055*). In patients 3 and 4, two heterozygous variants were found in the ALMS1 gene (c.7447A>T, p.Lys2483* and c.10930C>T, p.Gln3644*), confirming the diagnosis of Alström syndrome. These variants had not been previously described in the medical literature and represent a new finding in the understanding of this rare syndrome.

Because Alström syndrome is a complex disease that affects several organs, with a gradual unfolding of phenotypes, it is difficult to establish an early diagnosis. The variability of clinical presentations, even in patients from the same family, who carry the same mutation, suggests that unknown genetic or environmental modifiers probably interact with ALMS1. Therefore, the in-depth knowledge of new diagnosed cases and the report of new variants become fundamental to help the clear understanding of AS pathogenesis, aiming to improve the quality of life and prolong the survival rates in patients with this rare syndrome.

## Conclusion

Alström syndrome can affect multiple organs and manifest differently throughout the patients. Our detailed descriptions of these four cases and their phenotypes enable us to deepen the knowledge about this rare disease, with diagnostic and prognostic implications. The audiological evolution of patients with familial Alström syndrome demonstrated the occurrence of slowly progressive bilateral sensorineural hearing loss, starting in early childhood, but with normal neonatal hearing screening. This reinforces the need for audiological follow-up throughout the development of these patients. In our study, two heterozygous mutations in the ALMS1 gene were also identified in two patients from the same family. The two variants found have not been previously described in the literature, which expands the spectrum of ALMS1 variants in Alström syndrome.

## Funding

This research did not receive any specific grant from funding agencies in the public, commercial, or not-for-profit sectors.

## Conflicts of interest

The authors declare no conflicts of interest.
